# Editorial: The role of the brainstem and cerebellum in autism and related neurodevelopmental disorders (DD)

**DOI:** 10.3389/fnint.2022.957003

**Published:** 2022-08-31

**Authors:** Eric London, Rubin Jure, Patricia Gaspar, Luis Puelles, Randy J. Kulesza

**Affiliations:** ^1^Institute for Basic Research in Developmental Disabilities (IBR), Staten Island, NY, United States; ^2^Centro Privado de Neurología y Neuropsicología Infanto Juvenil WERNICKE, Cordoba, Argentina; ^3^INSERM U839 Institut du Fer à Moulin (IFM), Paris, France; ^4^University of Murcia, Murcia, Spain; ^5^Lake Erie College of Osteopathic Medicine, Erie, PA, United States

**Keywords:** autism, brainstem, cerebellum, developmental disorders, hindbrain, midbrain

An often-told story: One night, a man going into his favorite tavern saw a friend of his on all fours on the street in front of the tavern. “What are you doing?” “I lost my key” “Good luck finding it”. Two hours later the man is leaving the tavern and his friend was still on all fours in the same place. “What are you doing?” “I lost my key” “I know, but you have been looking in the same place for 2 h it is not likely to be there” “I know, but I am under the only streetlight”. Similarly, much of brain research chooses to look under the streetlight rather than where the key may be. The cortex and to some extent other forebrain structures are well lit. Large, near the surface, amenable to various types of scanning, imaging, and electrophysiology. The Brainstem until recently has been difficult to study in humans due to its small size, difficult to access location, and densely packed cells which have exuberant connectivity to all parts of the brain. As a phylogenetically persevered structure, it might seem that its role in a disorder of higher functioning which is unique to the human is unlikely. Recent as well as time-tested research, however, contradicts this, as hindbrain structures are known to be a crucial participant in nearly all cognitive functions. The brainstem, which is functioning early in fetal life, and is largely developed at birth, is crucial in the development of the higher centers (Joseph, 2000; Kohlmeier and Polli, 2020). Although once considered “hard wired” at birth, this isn't the case and the brainstem can exhibit high levels of plasticity itself (Kohlmeier and Polli, 2020). Speaking developmentally, cortical development could not proceed typically if there was aberrant functioning on the level of the hindbrain and midbrain structures. Despite research labeling various functions as belonging to some cortical area, in reality, the circuits must include the brain stem and cerebellum. As the pathology and signs and symptoms of ASD and related developmental disorders remain largely unexplained, much like the lost key, we must broaden our search. The call for this special topic was generated by the desire to stimulate interest and research which includes these underexplored areas.

Of the midbrain and hindbrain structures, the cerebellum is the most studied in ASD and there is a growing literature to document cerebellar abnormalities in ASD. The role of the cerebellum in higher cognitive functions is no longer disputed. There is literature on the cerebellum's role in higher cognitive functioning dating back to the mid-19th century (Wang et al., 2014). Cerebellar abnormalities are among the most frequently reported findings in ASD and treatment directly aimed at cerebellar functioning is being explored (Stoodley et al., 2017). In this special topic, two studies expand on the role of the cerebellum in ASD. One endophenotype which has been advanced is the ability to integrate past experience into the predictive process in order to facilitate behavior including the complex behaviors needed for social skills. The role of the cerebellum in mediating these functions is reviewed in those with and without ASD by Frosch et al. Sensorimotor functioning is often not easily observable and also could be endophenotypes in ASD. Here McKinney et al. found increased force variability and saccade errors which correlated with localized cerebellar volumes.

Rather than conceptualizing ASD as a set of localized anomalous circuitry, many of the symptoms can be explained by deficits in neuromodulation (London, 2018). The brain's neuromodulators (dopamine, norepinephrine, serotonin, etc.) are centered in the brainstem their major centers such as the locus coeruleus, the ventral tegmental area, the substantia nigra, and the raphe nuclei. These areas have an extensive role in both brain function and development. Most of the medications which are used in psychiatry and in ASD mediate these neuromodulators. Dopamine neuron distribution and neurotransmission anomalies have been shown in several ASD animal models. Using valproic acid in embryonic domestic chicks, Adiletta et al., report that there is a rostro-caudal redistribution of dopamine neurons in the mesencephalon along with expression of genes linked to dopamine function in the septum, an area associated with social behavior. The Locus Coeruleus (LC) norepinephrine system has a growing body of literature to suggest its role in ASD. Keehn et al. report deficits in disengagement compared to typically developing peers, and this deficit was associated with increased resting pupil diameter suggestive of increased tonic activation of the LC.

The most highly developed literature on the brainstem in ASD pertains to the auditory system. A comprehensive review of the auditory and vestibular system by Mansour et al. with both anatomic and functional evidence makes a strong case for impairments in both systems at birth, and calls for tests of these two systems for as screenings to be done neonatally. The same group (Mansour and Kulesza), expanding on their previous work in which they found significant changes in neuron number and drastic dysmorphology in the superior olivary complex, found no changes in the Ventral Nucleus of the Trapezoid Body (VNTB) in ASD or the Valproate animal model. Here, they extended their work to show no changes in the ascending or descending tracts of the VNTB. Using the reports of sound localization deficits in ASD, which could lead to difficulty in the interpretation of complex sounds (language), Chokr et al. offer a hypothesis concerning the possibilities of the ASD-linked genes to cause developmental impairment of the Medial Nucleus of the Trapezoid Body. Möhrle et al. using the hypothesis of the excitation to inhibition ratio, and the Cntnap2 rat, measured auditory reactivity, filtering, and gating. They then treated with R-Baclofen (a GABA B agonist) and as a result, suggested that this medication could be useful for targeting sensory processing deficits in ASD. Chawla and McCullagh, reflecting on sensory sensitivities in the auditory system, specifically in Fragile X (in a knockout mouse), found that there is a deficit in high-frequency hearing and also an increased latency in the binaural interaction component in males, which is necessary for sound localization and could affect the processing of information.

Two papers suggest clinical tools for studying the brainstem in ASD. With the hypothesis of the centrality of brainstem functioning in ASD, Burstein and Geva present a review of brainstem-related markers which can be studied in early life. This would be able to anticipate the later occurring signs and symptoms which we are now using. These brainstem-related findings could serve to facilitate screening, prevention, and intervention. Acknowledging the difficulties in visualizing the brainstem and cerebellum, and therefore our inability to study brainstem in children (Guerrero-Gonzalez et al.) present a new scanning methodology which yields better visualization, improved microstructural property measurements, thus opening up the way for the study of the brainstem in ASD.

Two broad-based reviews of the role of the brainstem in ASD are in this special topic. In a systematic review of this topic, Seif et al. offer post-mortem studies, functional, neuroimaging, auditory brainstem response, pupillometry and eye tracking and cardiovascular autonomic monitoring is included, in addition to an array of animal models. In a review focusing on neuropathologic evidence for the brainstem's role in ASD (Baizer), three functions found in ASD were focused on; (1) motor control, (2) auditory and vestibular information processing and (3) control of affect. In addition to reviewing many of the structures which have been written about in connection with ASD, she introduces some structures not commonly associated with ASD such as the Pontine Nuclei, the Arcuate Nucleus, and the Red Nucleus all of which could be key to understanding the circuitry involved in the communication between the cerebellum, cerebral cortex, and brainstem.

In a hypothesis paper (Jure) an extremely detailed account is given of how the Superior Colliculus' functioning is central to ASD. This paper creates a unique synthesis between an extensive array of clinical findings in ASD (well beyond the “core” symptoms), a list of many theories attempting to explain the findings in ASD, and the neuroscience background which can unify a great deal of these seemingly disparate findings and hypotheses. With so much evidence reviewed, the paper reminds us that these observations are yet to be tested with post-mortem or non-invasive functional human studies. One can easily observe that in a call for papers concerning the hind and midbrain's role in ASD, the majority of the papers are not original research. The goal of this special issue is to stimulate investigators to note the wealth of possibilities that exist if only they can find ways of searching for the key away from the streetlight.

## Author contributions

All authors listed have made a substantial, direct, and intellectual contribution to the work and approved it for publication.

## Conflict of interest

The authors declare that the research was conducted in the absence of any commercial or financial relationships that could be construed as a potential conflict of interest.

## Publisher's note

All claims expressed in this article are solely those of the authors and do not necessarily represent those of their affiliated organizations, or those of the publisher, the editors and the reviewers. Any product that may be evaluated in this article, or claim that may be made by its manufacturer, is not guaranteed or endorsed by the publisher.
